# Insulin Receptor Substrate 2 Is Required for Testicular Development

**DOI:** 10.1371/journal.pone.0062103

**Published:** 2013-05-31

**Authors:** Richard J. Griffeth, Jose Carretero, Deborah J. Burks

**Affiliations:** 1 Centro de Investigación Príncipe Felipe, Valencia, Spain; 2 Anatomy Department, University of Salamanca, Salamanca, Spain; 3 Centro de Investigación Biomédica en Red de Diabetes y Enfermedades Metabólicas Asociadas, Valencia, Spain; Clermont Université, France

## Abstract

Insulin receptor substrate (IRS) proteins are key mediators of insulin and insulin-like growth factor (IGF) signalling. In mice, deletion of *Irs1* is associated with profound growth retardation and increased longevity whereas *Irs2-*deficiency causes diabetes and female infertility. Clinical studies suggest that diabetes and obesity diminish male fertility. However, the role of IRS proteins in male reproduction is unknown. We observed that testis weight is reduced by 45% in *Irs2*-deficient mice as compared with control males. The weight of these organs in *Irs1* ^−/−^ males was similar to controls; however, since *Irs1*-deficient mice are 50% smaller, testis weight:body weight was increased in this model. Neonatal *Irs2* ^−/−^ mice also exhibited reduced testicular size, suggesting that impairments in this model occur during development. Histological examination of testicular cross sections from *Irs2* ^−/−^ mice revealed normal cellular associations without obvious abnormalities in the seminiferous epithelium. Reduced testicular weight was associated with fewer Sertoli cells, spermatogonia, spermatocytes, elongated spermatids, and epididymal spermatozoa. However, Leydig cell number and the concentration of serum testosterone were equivalent between *Irs2*-deficient and control males. Testicular weight was reduced similarly in non-diabetic and diabetic *Irs2* ^−/−^ mice, indicating that hyperglycemia does not compound the effects of *Irs2* deletion on impaired testis development. Expression of *Irs1*, *Irs3*, and *Irs4* was comparable between experimental groups. Collectively, our results demonstrate that IRS2 plays a critical role in testicular development, potentially by mediating IGF1 signalling during embryonic and early postnatal development.

## Introduction

In mammals, reproduction is controlled by the hypothalamic-pituitary-gonadal axis but is also modulated by changes in energy homeostasis and metabolism [1]. Insulin and its related growth factors, insulin-like growth factor (IGF) 1 and IGF2, promote proper metabolism, energy balance, and maintenance of normal body weight [2], [3], and modulate a variety of cellular activities including cell growth, proliferation, survival, and differentiation [4], [5]. These pleiotropic effects are tissue and development dependent [6], [7] and play a critical role in the regulation of reproduction and human embryonic development [8]–[10]. Insulin/IGF1 signalling is implicated in male reproduction; both are secreted by Leydig and Sertoli cells [7], [11]–[15], suggesting that they may modulate spermatogenesis [12], [14], [16], [17]. Consistent with this, receptors for IGF1 and/or insulin have been described at all stages of spermatogenesis in a variety of mammals [17]–[21]. Gene knockout studies have further demonstrated the importance of the insulin/IGF1 signalling pathway in growth and reproduction. Deletion of *Igf1* causes reductions in body weight, testis weight, testosterone concentration, and sperm counts [22]. In the absence of the insulin family of receptors, male sex determination during embryonic development is inhibited; the reproductive tracts of triple mutants deficient for *Ir, Igf1r, Insulin-related receptor (Irr)* resemble those of XX embryos and exhibit molecular profiles characteristic of females, indicating that testis determination requires insulin signalling [23].

Insulin and IGF1 activate their tyrosine kinase receptors in the plasma membrane [24], [25], which leads to tyrosine phosphorylation of numerous docking proteins including insulin receptor substrate (IRS) proteins [9]. Despite the structural homology between the IRS proteins, studies of knockout models have indicated that the various IRS proteins serve complementary rather than redundant roles in insulin/IGF1 signalling [26]. Loss of *Irs2* causes diabetes in mice due to beta cell insufficiency and peripheral insulin resistance [37]. Mice that lack *Irs1* display profound growth retardation but do not develop diabetes because insulin secretion compensates for the presence of mild insulin resistance [1], [27], [28]. There are minimal metabolic, endocrine, and growth phenotypes associated with deletion of *Irs3* or *Irs4* [29], [30].

Obese men and women are more likely to develop diabetes [31]. Furthermore, obesity and diabetes are associated with infertile conditions such as polycystic ovarian syndrome [12] and low sperm counts [32]. Studies in rodents suggest that diabetes decreases sperm counts and affects various aspects of male reproduction [33], [34]. Interestingly, a study in men with diabetes revealed that conventional semen parameters were not different between diabetic and non-diabetic men, however there were significantly more nuclear DNA fragmentation and mitochondrial DNA deletions in sperm from diabetic patients which may impair their reproductive capability [35]. The molecular mechanisms altered by diabetes in male reproduction have not been identified. Female *Irs2* null mice develop moderate obesity and are infertile due to hypogonadism [1], [36]. While insulin/IGF signalling pathways are clearly involved in reproduction, the effects of deletion of *Irs2* upon male reproduction have yet to be elucidated. Observations from the present study reveal that whereas *Irs1*-deficient males display an increase in testicular weight relative to body weight, testicular size is impaired in *Irs2* null mice, causing a 45% reduction of testis weight in adult animals.

## Materials and Methods

### Ethics Statement

Animal experimentation was performed according to the Guide for the Care and Use of Laboratory Animals of the National Institutes of Health (NIH Publication No. 85–23, revised 1996). The protocols for the study were approved by the Committee for Animal Welfare of Centro de Investigación Principe Felipe (registry ES 46 250 0001 002) which ensures compliance with national and European legislation regarding the use of animals in research.

### Animals

Mice were housed under a 12 h light/dark cycle at 23°C with access to food and water *ad libitum*. *Irs1* ^−/−^ and *Irs2* ^−/−^ mice were maintained on a C57Bl6J background. Mice were genotyped by PCR as described previously [37]. Fasting blood glucose levels were assessed by tail blood using an ELITE glucometer (Bayer Diagnostics, Barcelona, Spain). All adult mice used in these studies were 8–12 weeks of age. *Irs2* ^−/−^ male mice were grouped based on fasting concentrations of blood glucose: *Irs2* ^−/−^ ND (non-diabetic; blood glucose 90–110 mg/dl), or *Irs2* ^−/−^ D (diabetic; blood glucose >120 mg/dl).

### Histology and Immunofluorescence

Testes were immediately excised from euthanized animals, trimmed of fat and connective tissue, and weighed. One testis was snap frozen in liquid nitrogen and stored at −80°C until processed for protein or RNA extraction. The opposite testis was fixed in Bouin's Solution (Sigma, St. Louis, MO, USA) for 6 h, and then stored in 70% ethanol for 2–24 h, and embedded in paraffin. Testes from neonates were fixed in Bouin's Solution for 1 h.

Paraffin-embedded tissue was cut to a thickness of 5 µm, mounted on polysine slides, dewaxed, and rehydrated. For histology, tissue sections were stained with hematoxylin and eosin (Sigma). Images were captured using a Leica DM microscope (Switzerland) attached to Leica Application Suite (v 2.4.0 RI) imaging software. Assessment of morphological parameters was conducted using ImageJ software (v 1.43u, NIH, USA). For immunofluorescence, antigen retrieval was performed by incubating the slides in boiling 10 mM sodium citrate buffer (pH 6.0, Sigma) for 20 min. Non-specific binding was blocked by incubating the samples in blocking solution (1% BSA, 5% horse serum, 0.2% Triton-X in 1x PBS, Sigma) for 1 h at room temperature. Samples were incubated overnight at 4°C with one of the following primary antibodies: E-Cadherin (ECad), (Abcam #ab53033, 1∶100), proliferating cell nuclear antigen (PCNA), (Santa Cruz #56, 1∶100), DEAD-box protein 4/MVH (DDX4), (Abcam #ab13840, 1∶100), Nestin (Millipore #AB5922, 1∶300), or smooth muscle actin (SMA), (Abcam #ab5694, 1∶100). Samples were washed 5× in PBS and then the appropriate AlexaFluor conjugated secondary antibody (1∶400 Invitrogen, Eugene, OR, USA) was added and incubated in the dark for 30 min at room temperature. Samples were washed 4x in PBS, washed once in distilled water, then incubated with 1ng/ml 4,6-diamidino-2- phenylindole dihydrochloride (DAPI) for 5 min at room temperature and finally washed with distilled water. A few drops of Dako Fluorescent Mounting Medium (Barcelona, Spain) were placed on each slide and then covered with a coverslip. The slides were processed immediately and imaged using a Leica DM 6000B fluorescent microscope attached to Leica Application Suite (v 2.8.1) imaging software. All primary and secondary antibodies were diluted in blocking solution. Quantification of Sertoli cells, spermatogonia, spermatocytes, and Leydig cells was performed by counting cells positive by immunostaining in cross sections and verified by total cell counts of hematoxylin and eosin staining.

### Quantification of elongated spermatids

Elongated spermatids were estimated by the homogenization resistant spermatid technique as described by ÓDonnell L, et al. [38]. Briefly, after weighing one frozen testis, the capsule was removed and weighed and its weight was subtracted from the total weight of the testis to obtain the parenchymal weight. The parenchyma was placed in 1 ml PBS containing 0.05% Triton X-100 and homogenized with a polytron homogenizer (PT-MR 1600E, Kinematica Inc., Littau, Switzerland). An aliquot was taken and elongated spermatid heads were counted on a hemocytometer and expressed as spermatids per mg testis tissue.

### Sperm Capacitation

For individual mice, cauda epididymides from both testes were dissected free and rinsed in PBS before being placed in 1 ml TN medium (20 mM Tris (Sigma 25,285–9), 150 mM NaCl (Sigma S5886) and adjusted to pH 7.4). Using scissors, the epididymides were cut several times to allow sperm to passively exit. After 15 min, the remaining pieces of epididymides were removed and sperm in the TN medium were collected and counted on a hemocytometer. The TN medium containing sperm was transferred to a 15 ml conical tube and centrifuged at 800×*g* for 10 min at room temperature. The TN medium was removed and discarded and 2 ml of pre-equilibrated (overnight incubation at 37°C, 5% CO2 in a humidified atmosphere) capacitation medium (100 mM NaCl, 4.78 mM KCl, 1.71 mM CaCl2-2H2O, 1.19 mM KH2PO4, 1.19 mM MgSO4-7H2O, 23.6 mM sodium lactate, 0.11 mg/mg sodium pyruvate, 1 mg/ml glucose, 1% penicillin/streptomycin, 0.06 mg/ml phenol red, 20 mM Hepes, 20 mg/ml BSA, 2 mg/ml NaHCO3 and adjusted to pH 7.4) was carefully layered on top of the sperm pellet. This loosely-capped tube was placed in a vertical rack at 37°C, 5% CO2 in a humidified atmosphere for 2 h. The upper ml was removed from the conical tube and sperm motility and morphology from wild-type, *Irs2* ^−/−^ ND, and *Irs2* ^−/−^ D mice were visually assessed using a microscope.

### Protein Extraction and Western Blotting

Each frozen testis was placed in ice-cold lysis buffer at a concentration of 100 mg tissue per 1 ml lysis buffer and homogenized on ice using a polytron homogenizer. The lysis buffer contained 50 mM Tris pH 7.5, 200 mM NaCl, 0.2% NP-40, and 1% Tween-20. To detect 3βHSD RIPA buffer was used. The following components were prepared and added to the lysis buffer immediately before use, 50 mM β-glycerophosphate, 2 mM phenylmethylsulfonyl fluoride, 1 mM sodium orthovanadate, and 1x Complete protease cocktail inhibitor (Roche Diagnostics, Mannheim, Germany). The ice-cold lysis mixture was sonicated on ice three times for 5 sec and then incubated at 4°C for 30 min on a rotating wheel followed by centrifugation at 13,200×*g* for 15 min. Protein concentration was estimated using the BCA Protein Assay Kit (Thermo Scientific, Rockford, IL, USA). A total of 20 µg protein was loaded per lane and separated on 10% SDS-PAGE gels. Resolved proteins were then transferred to polyvinylidene fluoride (PVDF) membranes. Non-specific binding of proteins to the PVDF membranes was blocked by incubating in blocking solution containing 1x TBST –3% BSA for 1 h at room temperature. The PVDF membranes were then incubated overnight at 4°C with one of the following antibodies: IGF1R (Santa Cruz sc-713, 1∶1000), AKT (Cell Signalling #4691, 1∶1000), p-AKT (Cell Signalling #4060 1∶1000), ERK (Cell Signalling #4695, 1∶1000), p-ERK (Cell Signalling #4370), GSK3β (Invitrogen #44610, 1∶1000), p-GSK3β (Santa Cruz sc-11757 1∶1000), SOX9 (Abcam #26414 1∶1000), 3βHSD (Santa Cruz sc-28206 1∶500), or β-actin (Sigma #A1978, 1∶2000, 1 h at room temperature). Reactive bands were revealed using HRP-conjugated secondary antibodies (Thermo Scientific #31460 or #31430) with enhanced chemiluminescent reagents (Thermo Scientific) on X-ray film. Band intensities were quantified using Adobe Photoshop (v.CS2) and the intensity ratio for each protein was normalized to that of β-actin. Values obtained from testes of WT mice were set as 1 arbitrary densitometric unit.

### RNA isolation and quantitative RT PCR

Total RNA was extracted from whole frozen testes using TRIzol reagent (Invitrogen). RNA purification was achieved by treating samples with DNase I followed by the use of RNeasy Mini Kit (Qiagen, Hilden, Germany) and RNA concentrations were quantified using a nanodrop spectrophotometer. To generate cDNA, 1 µg of total RNA was reverse-transcribed on a thermocycler (Eppendorf, Madrid, Spain) using random hexamers in a total reaction volume of 20 µl under the following conditions: 25°C for 10 min, 42°C for 50 min, and 70°C for 15 min. Quantitative PCR was performed using a Roche Lightcycler 480 Real-Time PCR System machine (Barcelona, Spain) using the following TaqMan primers (Applied Biosystems): Gata1 Mm01352636_m1, Kit Mm00445212_m1, Ddx4 Mm00802445_m1, Androgen Receptor Mm00442688_m1, Hsd3b1 Mm00476184_g1, Irs1 Mm01278327_m1, Irs2 Mm03038438_m1, Irs3 Mm00802869_g1, Irs4 Mm01340253_m1, and Gapdh Mm99999915_g. PCR conditions were as follows: 3 min initialization at 94°C, followed by 40 cycles of 1 min denaturation at 94°C, 1 min annealing at 55°C, and 1 min elongation at 72°C. The target gene value was normalized to the expression of Gapdh. A negative control, without a prior reverse transcription reaction, was included for each gene. PCR data were analyzed by the comparative Ct method (2^−ΔΔCt^) [39]. Each reaction was performed in duplicate in four independent experiments.

### Quantification of Hormones

An enzyme-linked immunosorbant assay (USCN Life Science, Wuhan, China) was used to determine the concentration of testosterone in mouse serum according to the manufacturer's instructions. Each sample was analyzed in duplicate and values were extrapolated from a standard curve prepared with known concentrations of testosterone. Insulin and leptin were also measured by ELISA (Mercodia, Sweden).

### Statistical Analysis

Statistical analyses were performed using GraphPad Prism (v4.0). All data are presented as mean ± SEM. The differences in means were analyzed by ANOVA or unpaired t-test followed by Tukey's Multiple Comparsion Test or Welch's Correction, respectively. A difference of *P*<0.05 was considered statistically significant.

## Results

### Deletion of *Irs2*, but not *Irs1*, causes reduced testis size

**Figure 1:**
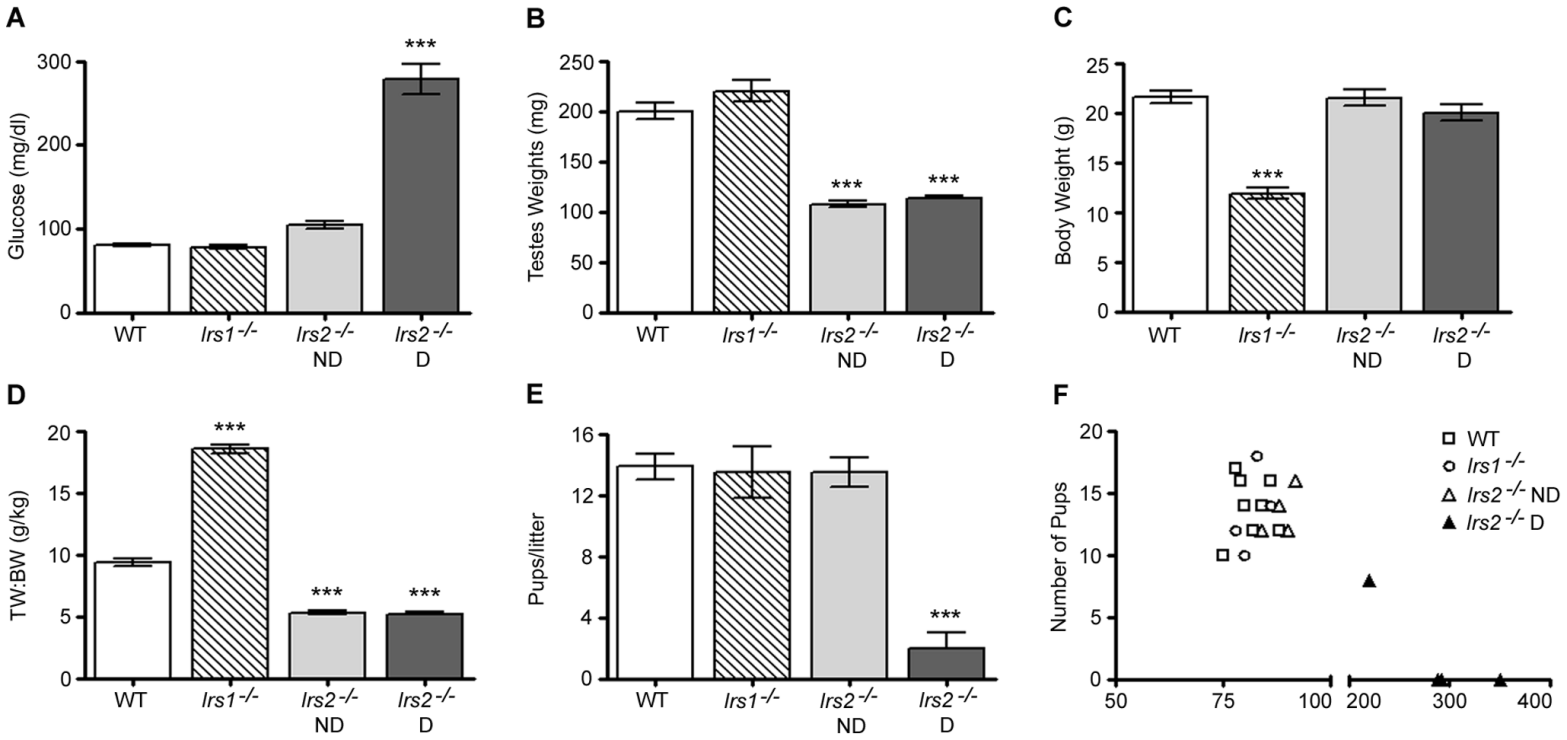
*Irs2*-deficient adult mice have reduced testis size and diabetic males father fewer pups. (A) Fasting blood glucose concentrations, (B) testes weights, (C) body weight, and (D) testes weights to body weight ratio were assessed in WT, *Irs1* ^−/−^, and *Irs2* ^−/−^ mice (n = 5 mice per experimental group). (E) To assess fertility, mating cages were established with two WT females and one male of the indicated genotypes. The number of pups per cage was monitored over a 4 week period. n = 4 males of each genotype. ND: mice with normal glucose levels; D: diabetic mice. (F) The number of pups/litter dramatically decreased with the onset of hyperglycemia (*Irs2* ^−/−^ D). Asterisks denote a significant difference compared to WT; *** P<0.001. All animals were 8–12 weeks of age.

Consistent with published results [1], [27], [37], some *Irs2* ^−/−^ mice became diabetic (D) around 10–12 weeks of age, whereas *Irs1* ^−/−^ mice exhibited normal blood glucose levels (Fig. 1A). *Irs*2-deficient mice were carefully monitored to permit the use of two experimental groups: *Irs2* ^−/−^ ND (non-diabetic; fasting blood glucose 90–110 mg/dl) and *Irs2* ^−/−^ D (diabetic; fasting blood glucose >120 mg/dl). Testes from both groups of *Irs2* ^−/−^ mice were reduced in size and weight but testes weights from *Irs1* ^−/−^ mice were not different than those of wild-type mice (Fig. 1B). However, when testes weights were normalized to body weight, the ratio of testes weights to body weight was significantly reduced in *Irs2* ^−/−^ mice compared to wild-type mice, whereas this ratio was significantly increased in *Irs1* ^−/−^ mice (Fig. 1C, D). Testes weights and the testes weight to body weight ratio were similar between *Irs2* ^−/−^ ND and *Irs2* ^−/−^ D mice, suggesting that the presence of diabetes in this model does not further reduce organ size.

The fertility of *Irs2* null males was comparable to wild-type males until the onset of hyperglycemia when reproductive capacity declined drastically (Fig. 1E, F). In the *Irs2*-deficient mouse model, diabetes develops due to reduced beta cell mass and insulin resistance, and most males die by 16 weeks of age due to a severe catabolic state as evidenced by weight loss, ketonuria and polydypsia [37]. As previously reported [1], [37], *Irs2*-deficient mice displayed elevated levels of both insulin and leptin (Fig. S1) until the onset of frank diabetes when beta cells are no longer able to compensate for peripheral insulin resistance. *Irs2* ^−/−^ males with hyperglycemia do not engage in normal rodent behavior such as grooming or exploration, related with weight loss and other derangements induced by uncontrolled diabetes. This low physical activity level presumably extends to mating behavior such as mounting since the generation of pups was greatly reduced in comparison to *Irs2* ^−/−^ ND when mated to fertile wild-type females (Fig. 1F).

**Figure 2:**
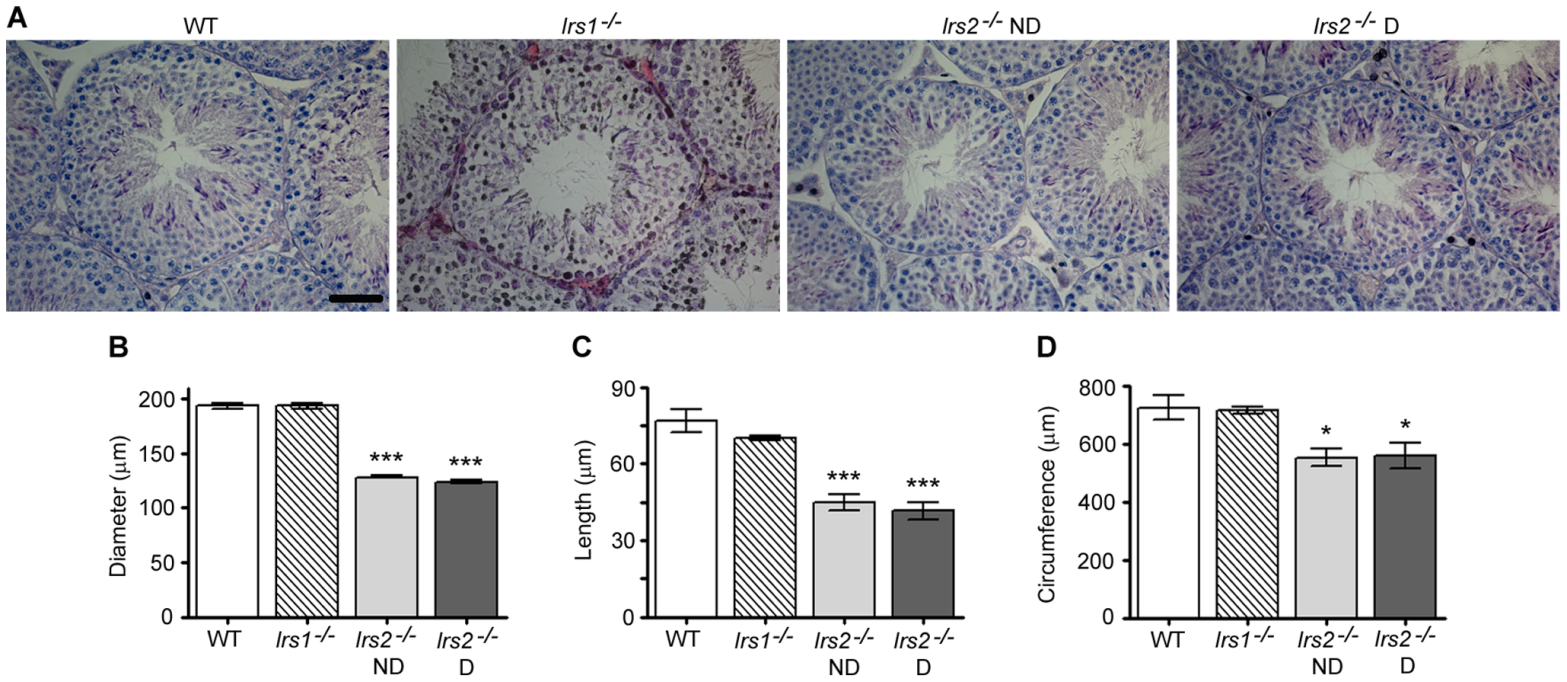
Testis morphology of *Irs2* ^−/−^ mice is normal but structures are reduced in size. Transverse histological sections of 5 µm were cut through the long axis of Bouin's-fixed paraffin-embedded testes. (A) Hematoxylin and eosin staining of testicular cross sections demonstrates normal cellular associations in the seminiferous epithelium and numerous elongated spermatozoa extending into the lumen. Representative images were captured using a 40x objective. The scale bar represents 50 µm. (B) The diameter of the seminiferous tubules, (C) the length of the seminiferous epithelium, and (D) the circumference of the seminiferous tubules were reduced in Irs2-deficient but not in *Irs1*-deficient mice. Results for each measurement are mean ± SEM from a minimum of 50 randomly selected seminiferous tubules from five mice from each phenotype. Asterisks denote a significant difference compared to WT; * *P*<0.05, *** *P*<0.001. Adult mice of 8–12 weeks of age were used for the study.

Histological examination of the testes by hematoxylin and eosin staining demonstrated that there were no apparent alterations in the seminiferous epithelium or interstitial spaces of testes from *Irs1* ^−/−^ or *Irs2* ^−/−^ mice (Fig. 2A). The seminiferous epithelium contained Sertoli cells and germ cells at all stages of differentiation and displayed apparently normal cellular associations (Fig. 2A). However, compared to wild-type mice, *Irs2* ^−/−^ mice exhibited significant reductions in the diameter of seminiferous tubules, the length of the seminiferous epithelium, and the circumference of seminiferous tubules. Results were similar between *Irs2* ^−/−^ ND and *Irs2* ^−/−^ D mice and there were no significant differences in these parameters between *Irs1* ^−/−^ mice and controls (Fig. 2B–D).

### Neonatal *Irs2*-deficient mice display reduced testes weights

**Figure 3:**
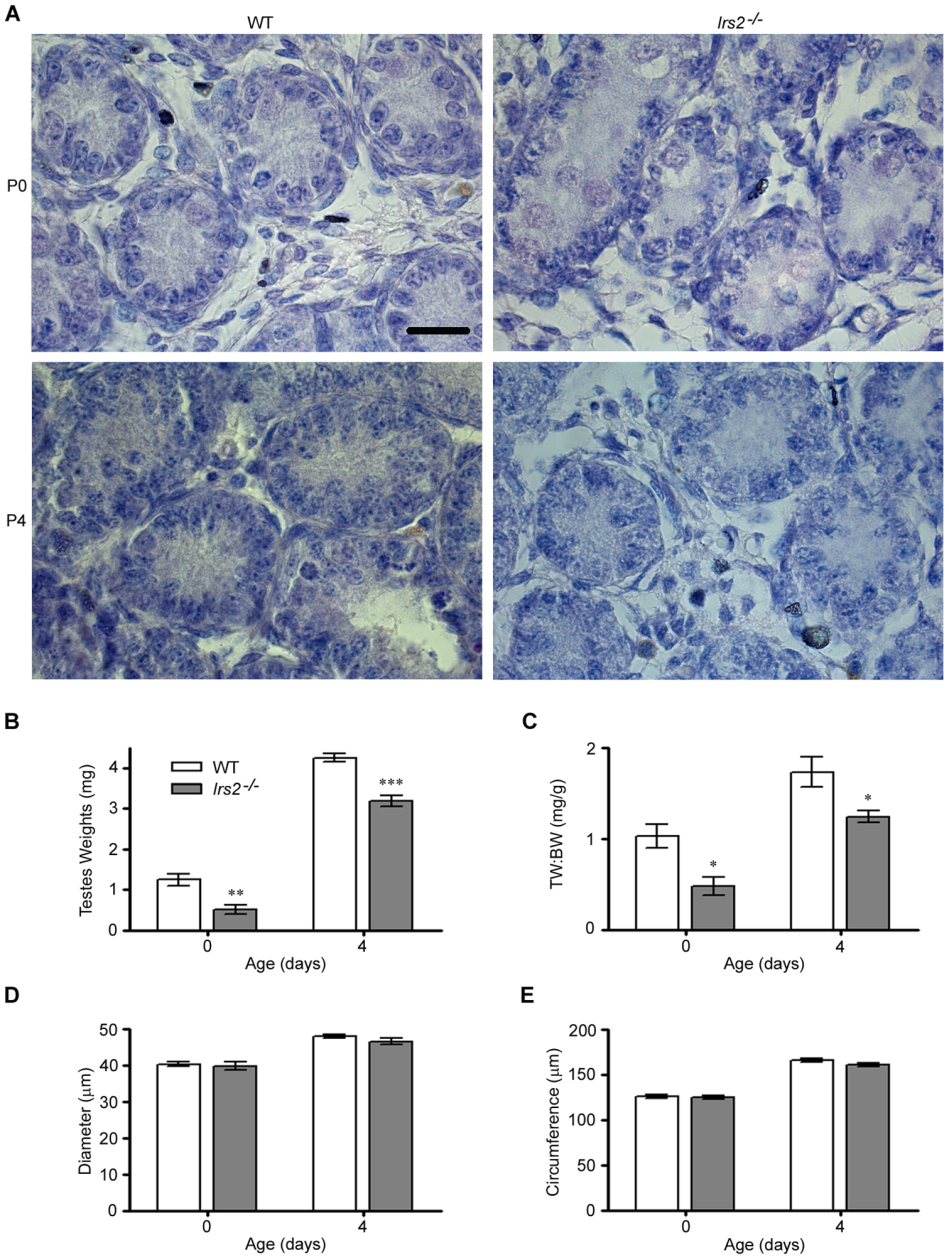
*Irs2*-deficient neonatal mice have reduced testis size. (A) Testes were fixed in Bouin's solution and embedded in paraffin, and sections were cut to a thickness of 5 µm and stained with hematoxylin and eosin. Images were captured at 100× magnification under oil immersion. The scale bar represents 20 µm. Representative images of testicular cross sections of WT and *Irs2* ^−/−^ at P0 (day of birth) and P4. (B) Testes weights and (C) the testes weights to body weight ratio were reduced in Irs2-deficient mice at P0 and P4. (D) Diameter and (E) circumference of seminiferous cords were measured in WT and *Irs2* ^−/−^ at P0 or P4. Asterisks denote a significant difference compared to WT; * *P*<0.05, ** *P*<0.01, *** *P*<0.001. (n = 4 male pups of each genotype).

To determine if the reduced testis size phenotype of *Irs2*-deficient adult mice reflects a developmental defect, testes from neonatal mice were analyzed at postnatal (P) day 0 (day of birth) and P4. Histological examination of testicular cross sections at P0 and P4 by hematoxylin and eosin staining revealed similar morphology between wild-type and *Irs2*-deficient pups (Fig. 3A). Testes weights of *Irs2* ^−/−^ pups at both time points were significantly reduced compared to controls (Fig. 3B) but no differences in body weight were noted. Therefore, when testes weights were normalized to body weight, the ratio was significantly reduced (Fig. 3C). There were no differences in the diameter (Fig. 3D) or circumference (Fig. 3E) of seminiferous cords at P0 or P4.

### *Irs2*-deficient mice exhibit reduced testicular cell number and sperm counts

**Figure 4:**
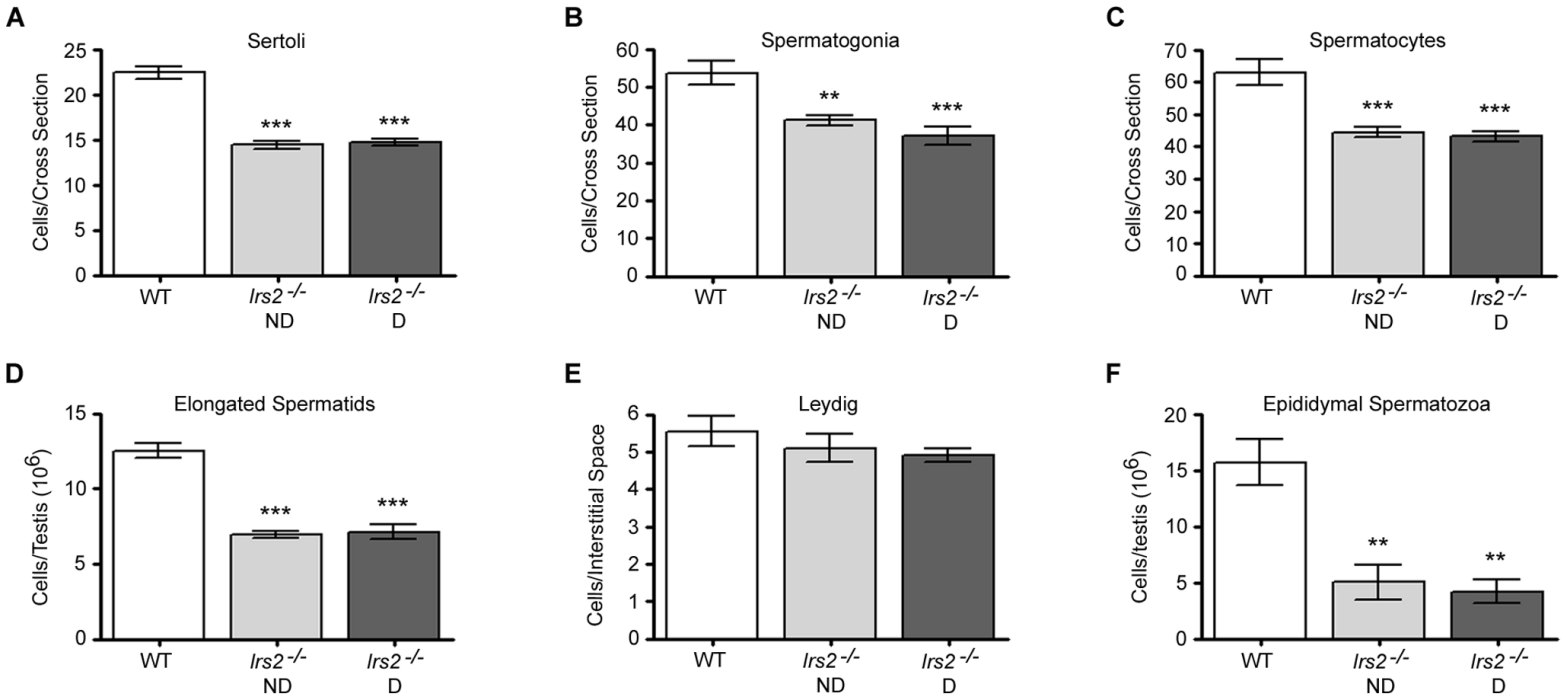
Analysis of distinct cell populations in the adult testis. (A–E) Transverse histological sections 5 µm in thickness were cut through the long axis of Bouin's-fixed paraffin-embedded testes (8–12 weeks of age) and sections were probed with antibodies to specific cell types which were visualized and quantified by immunofluorescence. (A–C) Cell number per cross section of seminiferous tubule was quantified in a minimum of 50 seminiferous tubules per mouse. (A) Sertoli cells were immunostained with anti-ECad. Results are mean ± SEM of 7 WT, 12 *Irs2* ^−/−^ ND, and 11 *Irs2* ^−/−^ D mice. (B) Spermatogonia were immunostained with an antibody to PCNA. Results are mean ± SEM of 10 WT, 11 *Irs2* ^−/−^ ND, and 10 *Irs2* ^−/−^ D mice. (C) Spermatocytes were immunostained with anti-DDX4. Results are mean ± SEM of 7 WT, 8 *Irs2* ^−/−^ ND, and 7 *Irs2* ^−/−^ D mice. (D) Elongated spermatids were quantified by the homogenization resistant spermatid technique and are expressed as the number of elongated spermatids/testis (10^6^). Results are mean ± SEM of 5 mice per phenotype. (E) Leydig cells were immunostained with an antibody to Nestin. A minimum of 50 interstitial spaces per mouse were examined. Results are mean ± SEM of 5 mice per phenotype. (F) Spermatozoa were collected from epididymides and quantified. Results are mean ± SEM of 5 mice per phenotype. Asterisks denote a significant difference compared to WT; ** *P*<0.01, *** *P*<0.001.

As germ cells mature during spermatogenesis, they are in constant contact with Sertoli cells in the seminiferous epithelium. The physical and metabolic support of Sertoli cells is required for germ cell differentiation, meiosis, and transformation into spermatozoa [40]. Testosterone is produced by Leydig cells and is required for proper spermatogenesis; therefore, maintenance of normal Leydig cell function is important for the reproductive capacity and fertility of males. To assess whether loss of *Irs2* alters specific cell populations and ultimately sperm production, multiple testicular cell types were quantified by immunohistochemistry (Fig. S2) and verified by hematoxylin and eosin. Significantly fewer Sertoli cells, spermatogonia, and spermatocytes per cross section of seminiferous tubule were detected in *Irs2* ^−/−^ mice compared to wild-type mice (Fig. 4A–C). Moreover, there were fewer elongated spermatids per testis in *Irs2*-deficient mice (Fig. 4D). Interestingly, there were no differences in the number of Leydig cells per interstitial space between wild-type and *Irs2*-deficient mice (Fig. 4E). Significantly fewer spermatozoa were collected from cauda epididymides of *Irs2*-deficient mice compared to controls (Fig. 4F). However, following *in vitro* capacitation, we detected no obvious abnormalities in morphology or motility between epididymal spermatozoa from wild-type or *Irs2*-deficient mice. No significant differences were observed in any cell population between the *Irs2* ^−/−^ ND and *Irs2* ^−/−^ D mice. Staining patterns of smooth muscle actin for peritubular myoid cells and collagen IV, a basement membrane structural component, were not different between wild-type and *Irs2* ^−/−^ mice (Fig. S3).

### *Irs2*-deficient mice have reduced cell number but normal cell density

**Figure 5:**
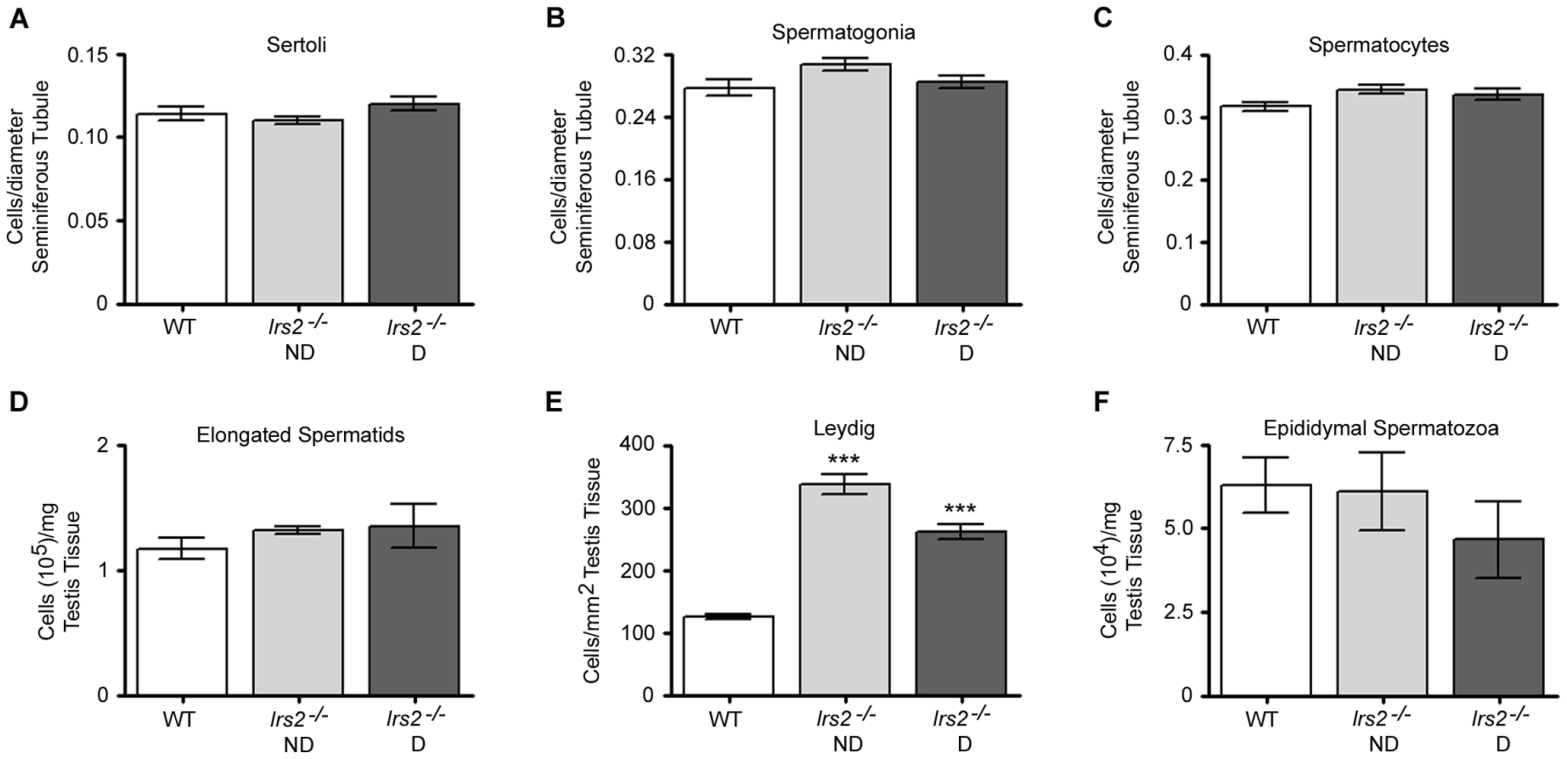
*Irs2*-deficient mice have reduced testicular cell number but normal cell density. For (A) Sertoli cells, (B) spermatogonia, and (C) spermatocytes, the number of cells per cross section of seminiferous tubule was normalized to the diameter of the seminiferous tubule. There were no differences between WT and *Irs2*-deficient mice (*P*>0.05). (D) The number of elongated spermatids was normalized to the weight of testis parenchyma. No significant differences were observed between WT and *Irs2*-deficient mice (*P*>0.05). (E) The number of Leydig cells was normalized to square area of testis tissue and there were significantly more Leydig cells per equivalent square area in *Irs2*-deficient testes compared to WT animals (*P*<0.001). (F) The number of spermatozoa collected from epididymides was normalized to testes weights. There were no significant differences between WT and *Irs2*-deficient mice (*P*>0.05).

Given the reduced size of Irs2-deficient testes, the quantification of cell populations was normalized. For Sertoli cells, spermatogonia, and spermatocytes, cell number was normalized to the diameter of the seminiferous tubule. After correction, there were no differences between genotypes (Fig. 5A–C). The number of elongated spermatids was normalized to testis weight and there were no differences between wild-type and *Irs2*-deficient mice (Fig. 5D). However, because there were more interstitial spaces per equivalent area, when the number of Leydig cells was normalized per square area of testis tissue, there were significantly more Leydig cells in testes of *Irs2* ^−/−^ mice (Fig. 5E). The number of spermatozoa collected from cauda epididymides was normalized to testes weights and no significant differences were observed between wild-type, *Irs2* ^−/−^ ND, or *Irs2* ^−/−^ D mice (Fig. 5F). Thus, although the testes of *Irs2* ^−/−^ mice are smaller and contain fewer cells than those of control mice, the general structure of these organs was normal.

### Loss of *Irs2* does not affect testosterone levels

**Figure 6:**
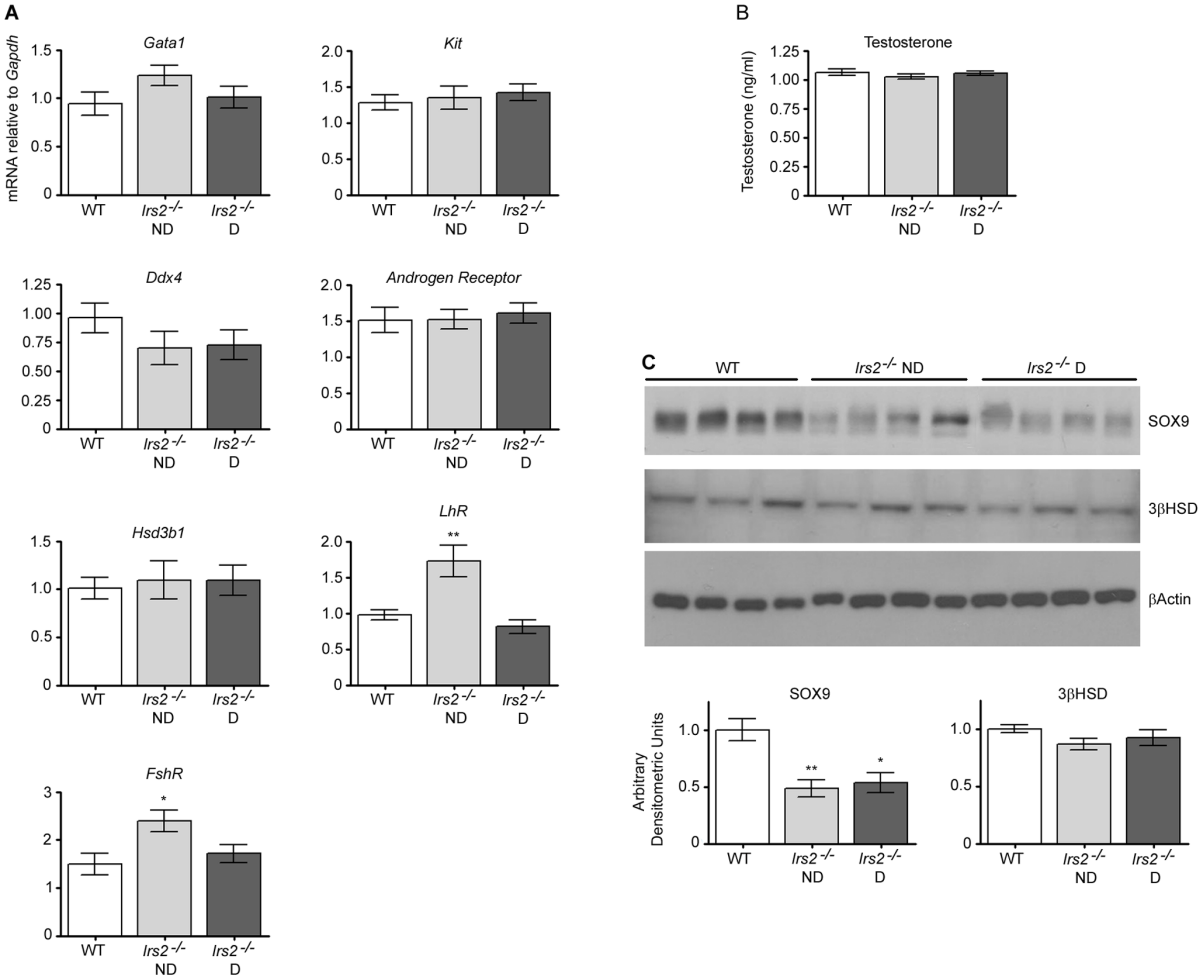
Analysis of cell-specific markers and serum testosterone in *Irs2*-deficient mice. Quantitative RT-PCR was performed with RNA obtained from testes (8–12 weeks of age) of the indicated genotypes. (A) Sertoli cell-specific marker *Gata1*, spermatogonial stem cell marker *Kit*, spermatocyte cell marker *Ddx4*, Leydig and peritubular myoid cell marker *Androgen receptor*, or Leydig cell marker *Hsd3b1.* Additionally, the receptors of FSH and LH were analyzed. (B) Testosterone in serum was measured by ELISA and no differences were seen between WT and *Irs2*-deficient mice (*P*>0.05). n = 4 mice per experimental group. (C) Immunoblots of SOX9 (Sertoli cell marker) and 3βHSD (Leydig cell marker) in the testis. Testes were homogenized in lysis buffer, 20 µg of total protein was loaded per lane, and blots were probed with an antibody against SOX9 or 3βHSD. β-actin was used as a loading control. The results are representative of three independent experiments. Band intensities were quantified using Adobe Photoshop (v.CS2) and the intensity ratio for each protein was normalized to that of β-actin. Values obtained from testes of WT mice were set as 1 arbitrary densitometric unit. Results are mean ± SEM of 4 mice (SOX9) and 3 mice (3βHSD) per genotype. Asterisks denote a significant difference compared to WT; * *P*<0.05, ** *P*<0.01.

Quantitative RT-PCR was performed to analyze gene expression of various markers in the testis. Relative gene expression of transcription factor and Sertoli cell marker *Gata1* [40], spermatogonial stem cell marker *Kit*, and spermatocyte cell marker *Ddx4* was not different between wild-type and *Irs2*-deficient mice (Fig. 6A). Testosterone exhibits its biological effects by binding to the androgen receptor which is localized to Sertoli and peritubular myoid cells [41]. The enzyme 3β-hydroxysteroid dehydrogenase (3bHSD/HSD3B1) converts 5-Androstene-3b,17b-diol to testosterone and is localized to Leydig cells. Expression of *Androgen receptor* and *Hsd3b1* was not altered in *Irs2*-deficient mice. Likewise, the concentration of testosterone in serum was not different between wild-type and *Irs2-*deficient mice, thereby excluding the possibility that diminished levels of male steroid hormones might contribute to reduced testis size (Fig. 6B). To determine if there were differences at the level of protein expression, Western blotting was performed using antibodies specific for Sertoli and Leydig cells. No differences were found in protein expression of the Leydig cell marker 3βHSD. However, *Irs2*-deficient mice exhibited reduced expression of Sertoli cell marker SOX9 (Fig. 6C).

### Loss of *IRS2* alters IGF1 receptor expression and basal activation of signalling pathways

**Figure 7:**
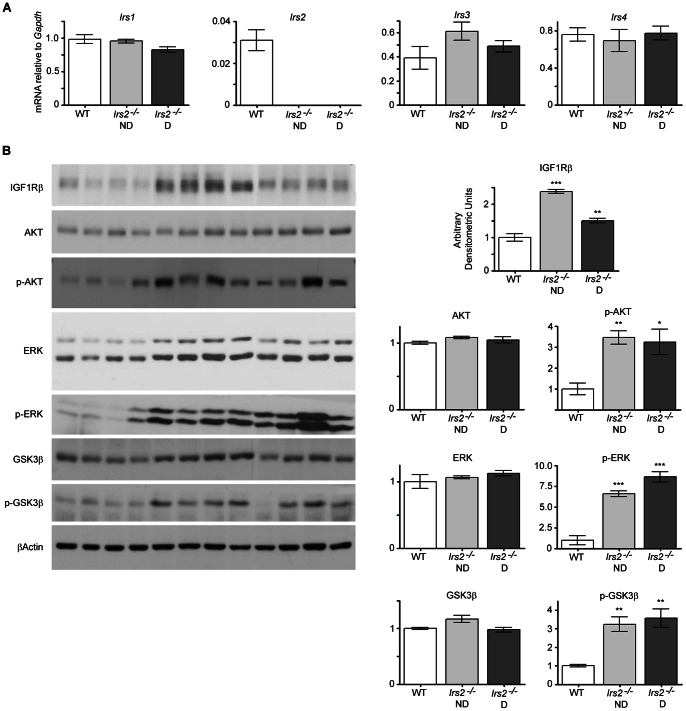
Insulin signalling in the testes of *Irs2*-deficient mice. (A) Gene expression of IRS proteins in the testes revealed that there were no differences in expression of *Irs1*, *Irs3*, or *Irs4* between WT and *Irs2*-deficient mice. Quantitative RT-PCR was performed using TaqMan probes. Each reaction was performed in duplicate and the value of the gene of interest was normalized to the expression of a control gene, Gapdh. For each gene, results are mean ± SEM of 5 WT, 5 *Irs2* ^−/−^ ND, and 6 *Irs2* ^−/−^ D mice. (B) Immunoblots of IGF1R, AKT, p-AKT, ERK, p-ERK, GSK3β, and p-GSK3β in the testis. Testes were homogenized in lysis buffer, 20 µg of total protein was loaded per lane, and blots were probed with the corresponding antibody. β-actin was used as a loading control. The results are representative of three independent experiments. Band intensities were quantified using Adobe Photoshop (v.CS2) and the intensity ratio for each protein was normalized to that of β-actin. Values obtained from testes of WT mice were set as 1 arbitrary densitometric unit. Results are mean ± SEM of 4 mice per phenotype. Asterisks denote a significant difference compared to WT; * *P*<0.05, ** *P*<0.01, *** *P*<0.001.

Since other IRS proteins have also been detected in the testis [42], we next determined whether loss of *Irs2* might alter expression of related proteins. There were no changes in gene expression of *Irs1*, *Irs3*, or *Irs4* between wild-type and *Irs2* ^−/−^ mice as detected by quantitative RT-PCR (Fig. 7A). Interestingly, the expression of the IGF1 receptor was increased in testes of *Irs2* ^−/−^ mice (Fig. 7B), perhaps indicating an attempt to compensate for failed IRS2 signalling in cells which are dependent on IGF1 regulation. Consistent with increased expression of IGF1R in *Irs2*-deficient testes, basal levels of p-AKT, p-ERK, and p-GSK3β were increased. Elevated basal PI-3 kinase activity has been previously reported for other tissues of the *Irs2* ^−/−^ model (37). The expression of AKT, ERK, or GSK3β was similar between *Irs2* ^−/−^ and control mice (Fig. 7B).

## Discussion

*Irs2*-deficient mice develop diabetes owing to reduced beta cell mass and peripheral insulin resistance [37]. Additionally, females of this model are infertile due to various defects in the reproductive axis [1], [36]. Signalling events downstream of IRS2 in the ovary play critical roles in follicular development and ovulation by regulating key components of the cell cycle which coordinate proliferation and differentiation [36]. However, the role of IRS proteins in male reproduction has not been investigated. The results of the present study reveal that deletion of *Irs2* causes a significant reduction in the size of the testis; this reduction of size is global since it does not target particular structures or cell populations in the testis but rather diminishes the components proportionally.

The role of IRS1 and IRS2 in testis function was examined initially by comparing wild-type, *Irs1* ^−/−^, and *Irs2* ^−/−^ mice. Visual inspection of male gonads suggested that mice deficient in *Irs2* had smaller testes and this was confirmed by weighing these organs (Fig. 1A). Testes weights were reduced by 45% in adult *Irs2*-deficient mice. However, testes weights of *Irs1*-deficient mice were not different than wild-type mice. Consistent with published studies, body weight of *Irs2* ^−/−^ mice was similar to wild-type mice, but body weight of *Irs1* ^−/−^ mice was significantly reduced [27], [37]. When testes weights were normalized to body weight, there were significant reductions between wild-type and *Irs2* ^−/−^, but not *Irs1* ^−/−^ mice, thereby confirming that deletion of *Irs2* but not *Irs1*, causes a specific reduction in the size of the testes (Fig. 1B). Neonatal testes weights at P0 and P4 were significantly reduced in *Irs2* ^−/−^ mice, suggesting that the reduced testis size phenotype is a developmental defect (Fig. 3B). Morphological measurements of the seminiferous tubules (Fig. 2B–D) and cords (Fig. 3D–E) verified these findings. These observations suggest a previously unknown role for IRS2 in testis development and reproductive function.

Various lines of evidence suggest that both type 1 and type 2 diabetes can lead to impaired male fertility in humans. Hyperglycemia has been shown to reduce the quality of sperm in male diabetic patients [35]. There are also other complications of diabetes that can severely impede the ability to reproduce including obesity, fatigue, loss of libido and the inability to maintain an erection [33]. The loss of *Irs2* causes diabetes in mice due to pancreatic beta cell insufficiency and most male *Irs2*-deficient mice die from diabetic complications by 16 weeks of age [37]. However, previous studies have demonstrated that *Irs2*-deficient males are adequate breeders, at least prior to the onset of severe diabetes [1]. To establish whether hyperglycemia compounds the effects of *Irs2*-deficiency on male reproduction, glucose levels were carefully monitored in this model. No differences were observed in testes weights between *Irs2* ^−/−^ ND and *Irs2* ^−/−^ D mice, suggesting that hyperglycemia does not further reduce testis size (Fig. 1B). Furthermore, subsequent analysis of testicular cell populations and expression of testicular markers revealed no differences between euglycemic and diabetic *Irs2* null mice, including the quantity of epididymal sperm. Thus, in the absence of IRS2 signals, spermatogenesis proceeds normally and produces morphologically normal spermatozoa, although the absolute numbers are reduced.

The reproductive capacity of *Irs2* males paired with wild-type females decreased drastically once hyperglycemia was detected (Fig. 1E, F), most likely as a consequence of the metabolic alterations of uncontrolled diabetes including weight loss, polydipsia, and polyuria. For species such as the mouse, the production of sperm far exceeds the number necessary for fertility. Quantitative spermatogenesis must be decreased by at least 90% in rodents to affect the numbers of progeny produced [43]. In *Irs2*-deficient mice, there was a 70% reduction in the total number of epididymal spermatozoa, although when normalized to testis weight the quantity of spermatozoa was not different than wild-type controls. Consistent with this, the *Irs2* ^−/−^ ND group successfully mated and produced pups at a similar rate to wild-type mice. As further evidence that *Irs2*-deficiency does not directly alter sperm function, there were no differences in morphology or motility between genotypes after *in vitro* capacitation of sperm samples. Combined with the results from the normalization of cell counts, these data indicate that spermatogenesis was functional in *Irs2*-deficient mice. However, progression to severe hyperglycemia led to changes in behavior, namely lack of physical activity, in the *Irs2* ^−/−^ D group which explains the precipitous decline in fertility as assessed by mating frequency and the number of pups produced when caged with wild-type females.

Microscopic inspection of testicular cross sections stained with hematoxylin and eosin revealed that, although each parameter was reduced in size, the organization and structural architecture of the seminiferous epithelium was intact with normal cellular associations (Fig. 2A). Interestingly, this global reduction of organ size without obvious effects on structural organization has also been reported in the brain and kidney: *Irs2*-deficient mice are born with a 35% reduction of brain size [44] and kidney size is reduced by 20% at P5 [45]. Although the brain and kidney are noticeably smaller in *Irs2*-deficient mice, all areas are present but reduced proportionally. Collectively, these observations regarding the developmental effects in testis, brain, and kidney suggest that IRS2 signals are indispensable for specific pathways during critical points of development in these organs.

Sertoli cells play a central role in testicular development as their number determines the size of the testes and the number of germ cells that can be supported during spermatogenesis [46]. In *Irs2* ^−/−^ mice, there were significantly fewer Sertoli cells and accordingly fewer spermatogonia and spermatocytes within cross sections of seminiferous tubules (Fig. 4A–C). However, when cell numbers were normalized to the diameter of the seminiferous tubules there were no differences between genotypes (Fig. 5A–C). During neonatal testicular development, testes weights increase as Sertoli cells proliferate [47] which occurs in mice from approximately E12.5 to P15 [48]. Subsequent increases in the diameter and length of seminiferous tubules are due to germ cell proliferation [47]. Interestingly, although there was no change in gene expression of Sertoli cell marker *Gata1,* protein expression of SOX9 was significantly reduced in testes of *Irs2*-deficient mice (Fig. 6C). Bioinformatic analysis has shown that various differentially expressed microRNAs target genes that are essential in mammalian gonadal differentiation including SOX9 [49], [50]. Expression of SOX9 is the first identifiable marker of the pre-Sertoli cell lineage in the embryonic testis. Thus, a decrease or delay in the expression of SOX9 during the period of Sertoli cell differentiation and proliferation would be expected to reduce the population of pre-Sertoli cells. Although we have observed a reduction of SOX9 in the adult testes of *Irs2*-deficient, further studies are required to determine the relation between IRS2 signals and SOX9 expression.

In contrast to Sertoli cells, there were significantly more Leydig cells in *Irs2*-deficient mice after data normalization because there were more interstitial spaces per equivalent area. However, protein expression of the Leydig cell marker 3βHSD was not altered, which is in accord with the normal level of serum testosterone observed in these males. Since testosterone concentrations in *Irs2*-deficient mice were similar to wild-type mice, spermatogenesis most likely proceeds normally, although the total number of spermatozoa is diminished proportionately to the reduced testicular weight.

IGF1Rs couple to IRS2 to mediate islet development during embryogenesis and postnatal growth [51]. *Igf1* and *Igf1r* are expressed in Sertoli and Leydig cells from E12-E18 during mouse development, with highest levels of both proteins detected at E18 [52]. Increased expression of the IGF1R was detected in adult *Irs2*-deficient testes (Fig. 7B). Taken together with the known role of IGF1 in gonad development [22], [53], [54], this observation suggests that loss of *Irs2* may delay or impair crucial phases of testicular development that are IGF1-dependent, which in turn may cause up-regulation of the IGF1R in an effort to compensate for loss of IRS2 signalling. *Irs1, Irs3,* and *Irs4* were not changed in testes of *Irs2* ^−/−^ mice (Fig. 7A), hinting that other IRS proteins do not compensate for the loss of IRS2 during testicular development. The increased basal phosphorylation of AKT and ERK, observed in testes of *Irs2*-deficient mice (Fig. 7B), has been previously reported in skeletal muscle and liver in the *Irs2*-deficient mouse model and may represent an important component of insulin resistance [51].

In summary, the results from these studies demonstrate that IRS2 plays an essential role in the regulation of testicular development. Although IRS1 exerts a critical role in general somatic growth, the present data implicate a specific role for IRS2 in the development of certain organs including the testis. Interestingly, reduced expression of *Irs2* has been detected in islets of patients with Type 2 diabetes [55]. Thus, our results provide important observations for further understanding the effects of defective insulin signalling and diabetes on male reproductive function.

## Supporting Information

Figure S1 **Fasting levels of serum insulin and leptin. n = 6 males of each experimental group.**
(TIF)

Figure S2 **Immunofluorescent localization of testicular cell types.** IF of (A) Sertoli cells using an anti-ECad antibody, (B) spermatocytes using an anti-DDX4 antibody, (C) Leydig cells using an anti-Nestin antibody, and (D) peritubular myoid cells using an anti-SMA antibody. Representative images from testicular cross sections from each phenotype are shown. All images were captured using a 40x objective and the scale bar represents 50 µm.
(TIF)

Figure S3 **Detection of collagen IV and smooth muscle actin in the basement membrane of testis sections.**
(TIF)
