# Supplementary figures and images for: Insulin Receptor Substrate 2 Is Required for Testicular Development
Source: PLoS One. 2013 May 31;8(5):e62103. doi: 10.1371/journal.pone.0062103 (PMC3669358; doi:10.1371/journal.pone.0062103)

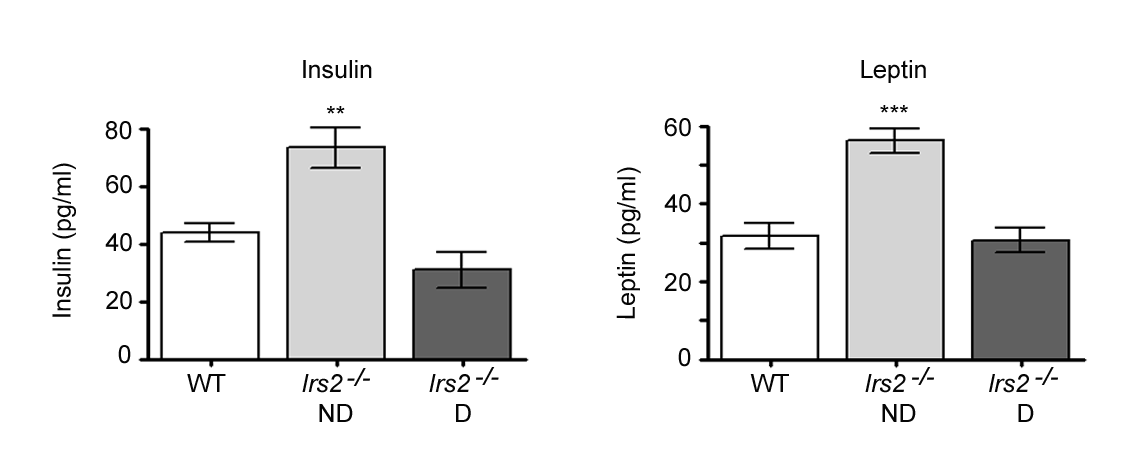

Supplement: Figure S1 — Fasting levels of serum insulin and leptin. n = 6 males of each experimental group. (TIF) [file pone.0062103.s001.tif]

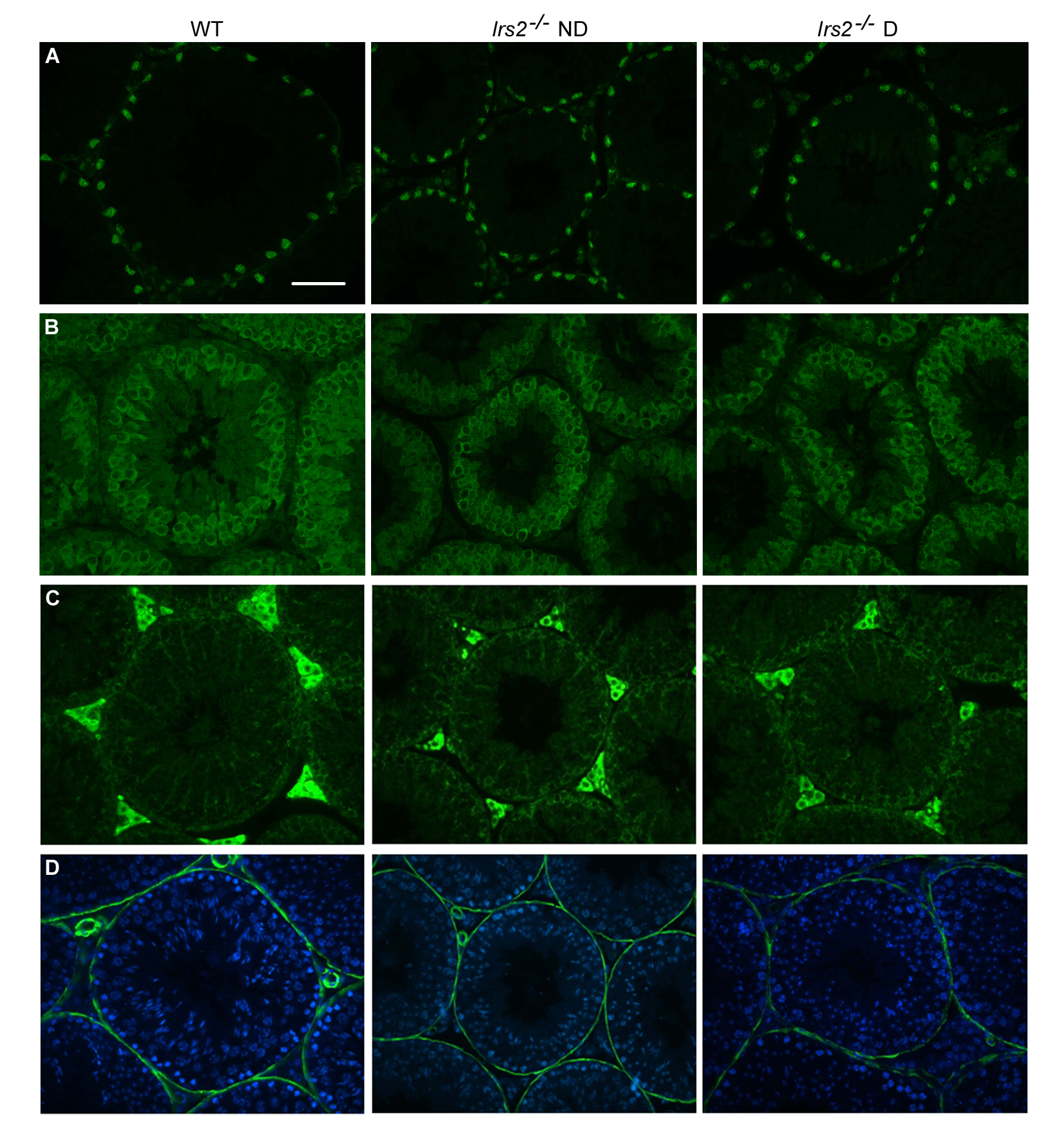

Supplement: Figure S2 — Immunofluorescent localization of testicular cell types. IF of (A) Sertoli cells using an anti-ECad antibody, (B) spermatocytes using an anti-DDX4 antibody, (C) Leydig cells using an anti-Nestin antibody, and (D) peritubular myoid cells using an anti-SMA antibody. Representative images from testicular cross sections from each phenotype are shown. All images were captured using a 40x objective and the scale bar represents 50 µm. (TIF) [file pone.0062103.s002.tif]

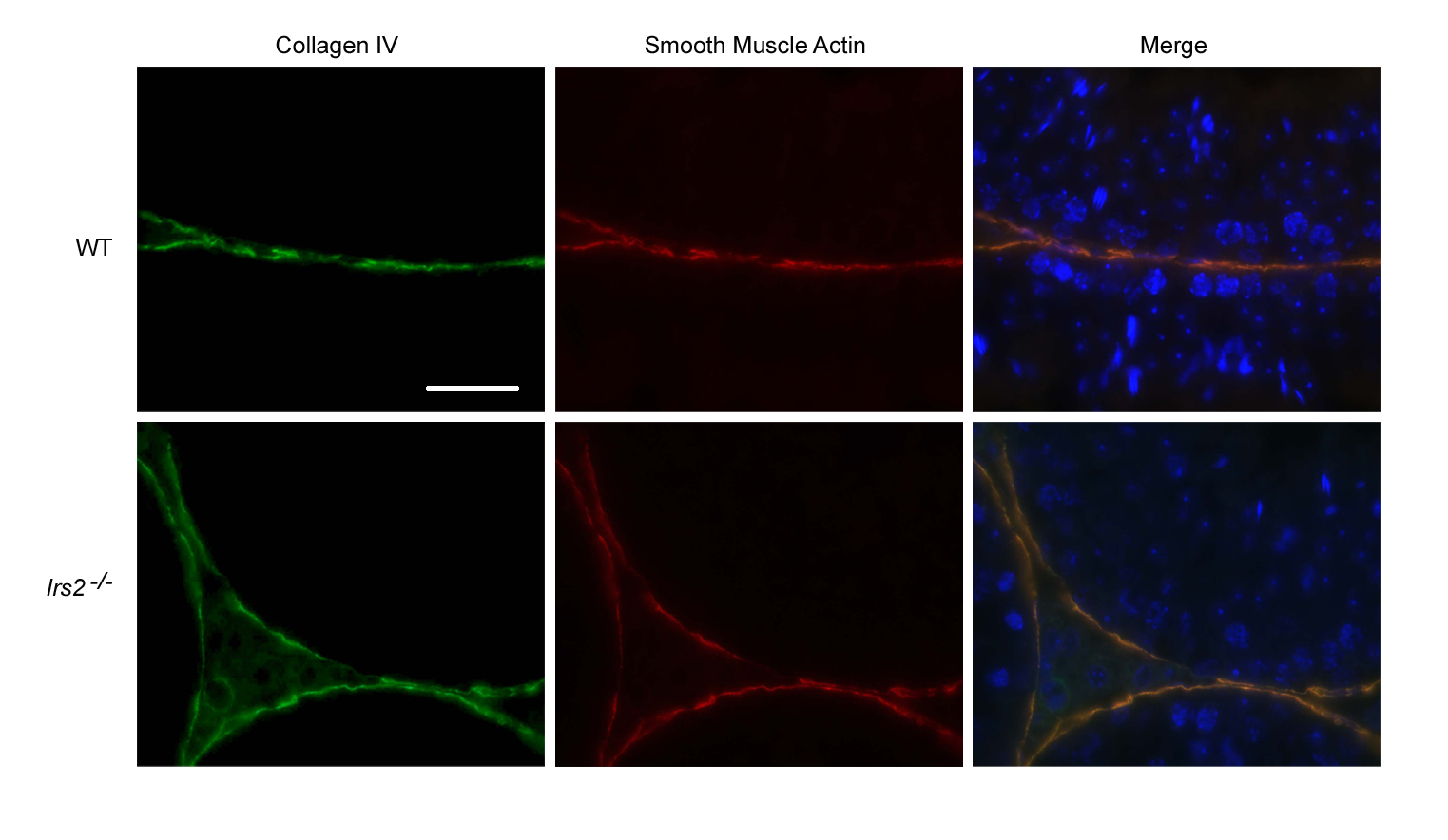

Supplement: Figure S3 — Detection of collagen IV and smooth muscle actin in the basement membrane of testis sections. (TIF) [file pone.0062103.s003.tif]
